# Central Nervous System Targets: Inhibitory Interneurons in the Spinal Cord

**DOI:** 10.1007/s13311-020-00936-0

**Published:** 2020-10-07

**Authors:** David I Hughes, Andrew J Todd

**Affiliations:** Institute of Neuroscience and Psychology, College of Medical, Veterinary and Life Sciences, University of Glasgow, Glasgow, Scotland

**Keywords:** GABA, glycine, spinal cord, chronic pain, allodynia.

## Abstract

Pain is a percept of critical importance to our daily survival. In most cases, it serves both an adaptive function by helping us respond appropriately in a potentially hostile environment and also a protective role by alerting us to tissue damage. Normally, it is evoked by the activation of peripheral nociceptive nerve endings and the subsequent relay of information to distinct cortical and sub-cortical regions, but under pathological conditions that result in chronic pain, it can become spontaneous. Given that one in three chronic pain patients do not respond to the treatments currently available, the need for more effective analgesics is evident. Two principal obstacles to the development of novel analgesic therapies are our limited understanding of how neuronal circuits that comprise these pain pathways transmit and modulate sensory information under normal circumstances and how these circuits change under pathological conditions leading to chronic pain states. In this review, we focus on the role of inhibitory interneurons in setting pain thresholds and, in particular, how disinhibition in the spinal dorsal horn can lead to aberrant sensory processing associated with chronic pain states.

**Electronic supplementary material:**

The online version of this article (10.1007/s13311-020-00936-0) contains supplementary material, which is available to authorized users.

## Inhibitory Interneurons in the Spinal Dorsal Horn

**Fig. 1:**
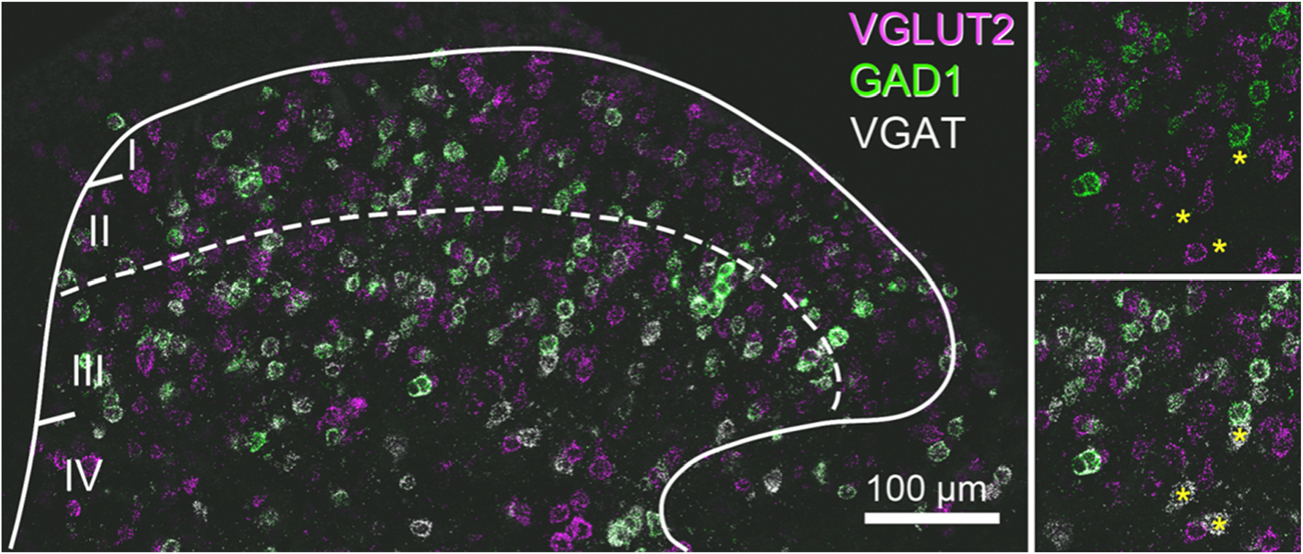
Neurotransmitter heterogeneity of dorsal horn neurons. Fluorescent *in situ* hybridization labelling for VGLUT2 (magenta), GAD1 (green), and VGAT (white) shows excitatory interneurons (magenta) outnumber inhibitory interneurons (green and white, or white only) in laminae I–III. VGLUT2-expressing interneurons are common in laminae I–III. Inhibitory interneurons can be split into three subpopulations based on their neurotransmitter content: those that express GABA, those that express glycine, and those that express both GABA and glycine. In this figure, inhibitory interneurons that only express GABA, and those likely to co-express both GABA and glycine, show co-expression for both GAD1 and VGAT (green and white, respectively) and are common in laminae I–III, whereas cells that express only glycine are common in lamina IV (insets, yellow asterisks). AM Bell, AJ Todd, and DI Hughes, *unpublished observations*

The dorsal horn of the spinal cord is the principal termination site of primary afferents that innervate the skin and deeper tissues of the trunk and limbs and is composed of several distinct classes of neurons. These afferent fibers engage discrete, modality-specific circuits comprised of spinal interneurons that play important roles in modulating and gating afferent input, and projection neurons that relay the processed information to higher brain centers [1]. Nociceptive afferents of various types terminate primarily in laminae I, II, and V, with the central terminals of thinly myelinated Aδ fibers terminating in lamina I and V [2], peptidergic C-fibers arborizing in lamina I and the outer part of lamina II (IIo), and non-peptidergic C-fibers that express the mas-related G protein-coupled receptor MrgD (CMrgD afferents) and bind isolectin B4 (IB4) terminating in mid-lamina II [3, 4]. Low-threshold mechanoreceptor afferents (LTMRs) terminate in deeper dorsal horn laminae, with unmyelinated C-LTMRs arborizing in the ventral part of lamina IIi, Aδ-LTMRs in lamina IIi and III, and Aβ-LTMRs in lamina IIi and III [5]. To allow the barrage to sensory input into the spinal cord to be perceived in context, afferent input into the central nervous system must be gated and prioritized—this process is achieved by the action of spinal interneurons. Local interneurons are thought to account for 99% of all neurons in the spinal dorsal horn [6] and can be subdivided into two principal classes based on their neurotransmitter content: excitatory interneurons that release glutamate and inhibitory interneurons that use GABA and/or glycine (Fig. 1). In both the rat and mouse, inhibitory interneurons account for approximately 25% of neurons in laminae I and II and 40% of those in lamina III [7–9]. These cells can be subdivided further into distinct subclasses based on their neurochemical, electrophysiological, and morphological properties [5, 10–15], but it has yet to be determined whether these represent functionally distinct populations. Given that the loss of inhibition in spinal circuits is thought to lead to aberrant processing of somatosensory input, the loss of pain suppression, and the development of a neuropathic pain-like state [16–18], these interneurons represent an obvious target for the development of novel pain management therapies. To facilitate this, we must first define the functional significance of various inhibitory interneuron subpopulations under normal conditions and then determine how the circuits contribute to change under pathological states that lead to chronic pain.

## Neurochemical and Molecular-Genetic Heterogeneity of Inhibitory Interneuron Populations

GABA acts as the major inhibitory neurotransmitter throughout most regions of the central nervous system, although glycinergic neurotransmission predominates in parts of the spinal cord and brainstem, and in the retina. Immunohistochemical studies in the rat and mouse show that GABA-immunoreactive (GABA-IR) cells in the spinal dorsal horn are concentrated in laminae I–III, whereas glycine-IR cells are rarely seen in laminae I and II, but are common in laminae III and IV [7, 9, 19–22]. In the rat, inhibitory interneurons account for between 25 and 30% of all cells in laminae I and II, and approximately 40% of those in lamina III [7, 8], with similar patterns being reported in the mouse [9]. Virtually all interneurons in laminae I–III that are enriched with glycine are also GABA-IR [7–9], and immunolabelling studies have shown that most axon terminals in this region that are derived from inhibitory interneurons contain both GABA and glycine [23–27]. This supports the view that axon terminals of most inhibitory interneurons in laminae I–III of the spinal dorsal horn co-release both neurotransmitters [28–30], but whether the resultant inhibition has both GABAergic and glycinergic components (distinguished pharmacologically) depends on the presence of corresponding neurotransmitter receptors at postsynaptic sites.

**Fig. 2:**
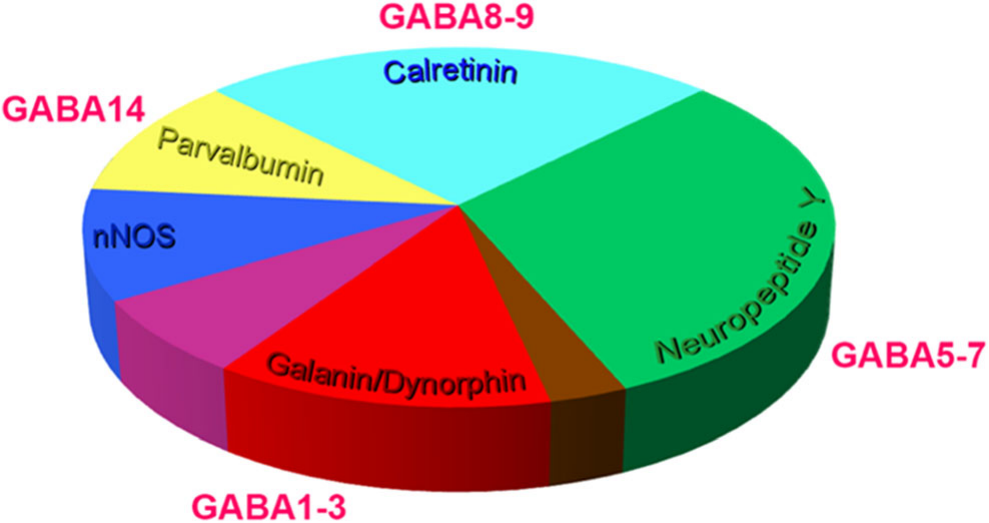
Neurochemical features of spinal inhibitory interneurons in laminae I and II. The estimated proportions of all inhibitory interneuron populations, as defined by their neurochemical profiles, are presented in the pie chart (modified from reference 10]. Four segments of this chart (parvalbumin, calretinin, neuropeptide Y, and galanin/dynorphin populations) correspond well with molecular clusters of inhibitory interneurons identified in single-cell RNA sequencing studies [12]. Taken together, these datasets provide a means of devising intersectional strategies to target subpopulations of interneuron with greater precision than possible previously

All dorsal horn inhibitory interneurons are believed to express the developmental transcription factor Pax2 [31–34]. Inhibitory interneurons in the superficial dorsal horn (laminae I–II) can be assigned to 5 largely non-overlapping populations (Fig. 2) on the basis of their expression certain neurochemical markers: the neuropeptides galanin and dynorphin (which are co-expressed), neuropeptide Y, neuronal nitric oxide synthase (nNOS), and the calcium-binding proteins parvalbumin (PV) and calretinin (CR) [10]. It is important to note that these markers are not exclusive to inhibitory interneurons, as several are also expressed by excitatory neurons (dynorphin, nNOS, calretinin, parvalbumin) or by primary afferents (galanin, parvalbumin). Recent studies of dorsal horn populations using open-ended genetic screening or transcriptomic approaches provide an unprecedented means of assessing the neurochemical and molecular-genetic profile of spinal interneurons [5, 11, 12]. The findings of one such study in the mouse identified 15 molecularly distinct subtypes of inhibitory neurons when single-cell RNA sequencing was used to classify dorsal horn neurons [12], and these largely match the neurochemically distinct populations identified in the superficial laminae using immunohistochemical approaches (Fig. 2), with the Gal/Dyn, NPY, CR, and PV populations corresponding to the Gaba1–3, Gaba5–7, Gaba8–9, and Gaba 14 clusters. Given that glycinergic populations cannot be identified with any great precision [12], this scheme is not definitive, but is nonetheless an important resource that provides a means of identifying unique molecular signatures in neurochemically defined neuronal populations. With the ever-increasing development of new transgenic mouse lines that express site-specific recombinases (SSRs), we can use such schemes to develop intersectional strategies in which co-expression of two recombinases (e.g., Cre and Flp), driven from different genes, is used to target specific neuronal populations [35, 36]. This will provide a means of targeting and manipulating neuronal populations with far greater precision than was previously possible.

## Morphological and Electrophysiological Features of Inhibitory Interneurons

Morphological heterogeneity among dorsal horn neuron populations has been a consistent finding, from early studies using Golgi labelling [37–39], to more recent studies where the morphology of individual cells was revealed following “blind” whole-cell recording in wild-type [13–15, 40, 41] animals and targeted recordings from transgenic mice [42–47]. The morphology of lamina II interneurons has been studied extensively. The most widely accepted scheme for classifying these neurons was developed from studies in hamsters [13], and defined four principal populations: islet, central, vertical, and radial cells, although ~ 20% of the neurons in their sample could not be assigned to any of these classes and were described as “unclassified.” Central cells were further subdivided into transient and tonic types, based on their action potential firing pattern in response to injected depolarizing current.

Similar morphological populations of lamina II neurons have been described in various species, including rat [14, 15, 40, 41, 48, 49] and mouse [42, 45, 46, 50]. It is still to be determined whether we can justifiably use morphological features alone as an indicator of whether cells are excitatory or inhibitory interneurons. A particular limitation is that most studies have used a purely subjective approach to assign cells to different morphological classes. Nonetheless, it is generally accepted that 3 of the classes identified by Grudt and Perl (radial, transient central, and vertical cells) in most cases correspond to subsets of excitatory interneurons, while islet cells are invariably inhibitory interneurons. However, it is also clear that many inhibitory interneurons in lamina II are not islet cells [46, 51].

**Fig. 3:**
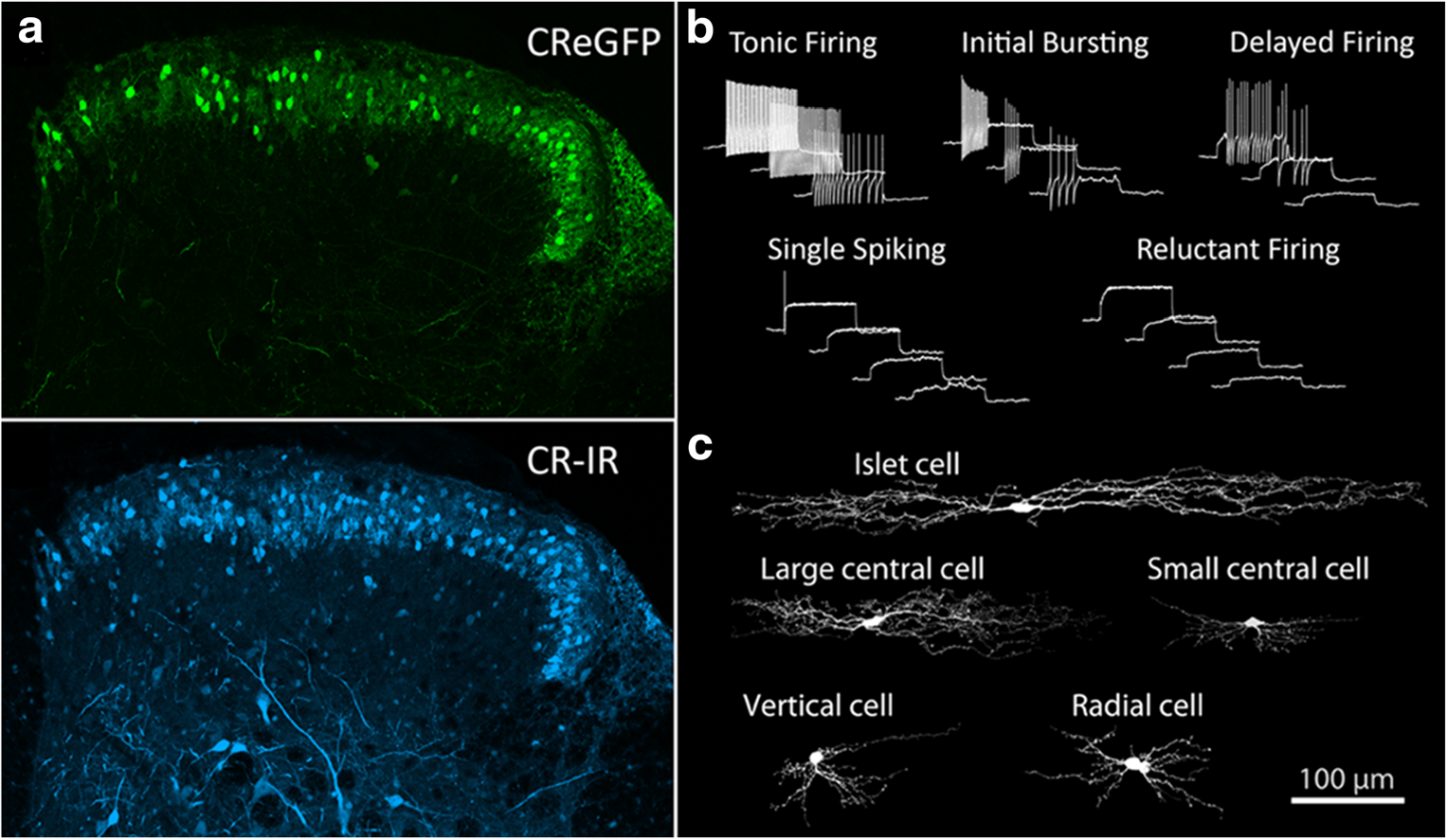
Morphological and electrophysiological diversity within a neurochemically-defined population lamina II neurons. Targeted whole-cell patch-clamp recordings were carried out in spinal cord slices maintained *in vitro* from a transgenic mouse line where enhanced green fluorescent protein (eGFP) was expressed in calretinin (CR) interneurons. (**a**) The distribution of eGFP-labelled cells mirrors that of calretinin cells labelled using immunohistochemical approaches (CR-IR). (**b**) Five distinct action potential firing patterns were recorded in eGFP cells from lamina II. (**c**) The morphological features of recorded neurons were also determined, with five morphologically distinct groups being recorded, as well as a group of unclassified cells. The only correlation between morphology and firing pattern that could be established was that cells with tonic-firing discharge patterns were always islet cells (and all islet cells were tonic firing). Modified from reference 45

Given that a variety of markers commonly used to define inhibitory interneurons in the dorsal horn can also be expressed in glutamatergic interneuron populations [5, 12, 45], using the expression of only a single neurochemical marker to identify neuronal populations can be misleading. The most widely used scheme for defining spinal interneurons is based on a system that combines the morphological and physiological properties of individual cells [13]. In this study, five morphologically distinct populations (islet, central, radial, vertical, and unclassified) were proposed, and three principal patterns of action potential firing were identified, namely tonic-, transient-, and delayed-firing discharge. Similar firing patterns have been described in the rat and mouse spinal cord [43, 50, 52, 53], with five distinct patterns being reported, namely tonic-, delayed-, and initial burst-firing, along with single spiking and phasic bursting. The incidence of cells displaying particular discharge properties appears to be correlated to the lamina in which the recordings were performed [52] and also on the holding potential used during these experiments [43, 53], but certain morphologically defined populations also appear to associate more commonly with certain firing patterns. For example, islet cells typically display tonic- or initial burst-firing action potential discharge patterns in response to depolarizing current injection steps, whereas radial cells, central cells, medial-lateral cells, vertical cells, and those cells of unclassified morphology displayed a range of other firing patterns including transient-, delayed-, and single spike-firing [13, 43, 45]. Several other studies have also reported similar correlations between firing patterns and morphology [15, 33, 40, 42], and these have helped propagate a general consensus that tonic- or initial burst-firing discharge patterns in representing recordings from inhibitory interneurons, whereas transient-, delayed-, and single spike-firing patterns are indicators of excitatory interneurons. Attempts to further define what appear to be homogenous populations by incorporating additional descriptive criteria can, however, be problematic. For example, calretinin-expressing cells (CRINs) in the spinal dorsal horn are largely confined to lamina II and have been considered to represent a population of excitatory interneurons [54]. More recently, interrogation of this neurochemically defined population of cells using immunohistochemistry, targeted whole-cell patch-clamp recordings, and transcriptomics have revealed that CRINs are morphologically, neurochemically, and physiologically diverse [12, 45, 55, 56] and display features found in both excitatory and inhibitory populations (Fig. 3). It therefore remains to be determined precisely which combinations of features are reliable indicators of transmitter content for other cell populations, and whether these morphological and electrophysiological signatures also apply to spinal neurons in other laminae.

## Spinal Inhibition

Inhibitory interneurons in the spinal cord can generate two distinct forms of synaptic inhibition mediated through the release of GABA and/or glycine and activation of ligand-gated ion channels (GABA_A_ and glycine receptors, respectively). Presynaptic inhibition is a GABA-mediated event resulting principally from the release of transmitter from axons (or presynaptic dendrites) that synapse with primary afferent terminals to act on GABA_A_ receptors primarily [57–60], although GABA_B_ receptors have also been implicated in this form of inhibition on both group Ia muscle afferents and Aβ cutaneous afferents [61, 62]. Postsynaptic inhibition results from the release (or co-release) of GABA and/or glycine at axodendritic and axosomatic synapses (primarily). While most inhibitory synaptic events typically have both GABA- and glycine-mediated components [60, 63–65] similar responses resulting from purely GABA- or glycinergic transmission have also been reported [66–68], and these support anatomical studies where axon terminals showing immunolabelling for only GABA or glycine have been described [24, 26, 69–71]. The presence of functional neurotransmitter receptors in the postsynaptic membrane will dictate the type of inhibition mediated at any given synapse, and the kinetics of these responses may also be highly dependent on the stoichiometry of receptors found at particular synapses [18, 72, 73]. Immunohistochemistry for the β3 subunit has been used to visualize GABA_A_ receptors at synapses [25, 27], whereas the microtubule-associated protein gephyrin (which anchors to the β glycine receptor subunit to the underlying cytoskeleton) is commonly used to visualize glycine receptor expression [25, 74–77]. The β3 subunit of the GABA_A_ receptor and gephyrin colocalize extensively at synapses formed by axon terminals containing both GABA and glycine [25, 27], but given that gephyrin-expressing synapses can also be found associated with axons enriched only in GABA [25, 27, 75], it is now widely considered to be a reliable marker of most inhibitory synapses in laminae I–III. The one notable exception to this generalized rule are axoaxonic synapses. These types of synapses are found on the central terminals of most types of primary afferents, with the exception of peptidergic C-fibers [78], and show immunolabelling for GABA_A_ receptor subunits but not for gephyrin [79, 80]. A large-scale single-cell RNA sequencing of dorsal root ganglion neurons also shows very low expression levels for the genes that encode for gephyrin (GPHN), or for any of the splice variants of glycine receptor α subunits (GLRA1–4) that supports the view that primary afferents do not express functional glycine receptors [81], as implied in earlier studies where glycinergic membrane currents could not be shown in dorsal root ganglion neurons [82]. Furthermore, studies using optogenetic approaches to define the pharmacological basis of presynaptic inhibition in both myelinated LTMR afferents (A-LTMR) from the skin and of proprioceptive afferent groups have demonstrated that this form of inhibition is insensitive to strychnine but can be abolished by bicuculline [60, 83], whereas light-evoked postsynaptic inhibition mediated in unidentified neurons were sensitive to both antagonists. Taken together, these findings imply that the action of both transmitters at specific synapses serve important, but as yet largely undefined, roles in the resultant inhibition, although mechanisms where these transmitters tonically inhibit inhibitory interneurons in laminae I–III of the spinal dorsal horn have been proposed as being important in separating low-threshold mechanoreceptive information from pain circuits in lamina I [84].

At the ultrastructural level, the central terminals of both non-peptidergic C-fibers and Aδ down hair afferents display distinctive anatomical features where they form the central elements of Type I and Type II glomeruli, respectively [85–87]. These glomerular arrangements provide structural insights into how the synaptic circuitry of the spinal dorsal horn is arranged to provide stringent control over the passage of sensory information into the central nervous system. The central terminals relay afferent input to spinal neurons by the release of glutamate at axodendritic synapses, and like most classes of primary afferents, are subject to presynaptic inhibition via axoaxonic synapses. Ultrastructural studies using post-embedding immunogold labelling have shown diversity of transmitter content within individual axon terminals that synapse on to both spinal neurons [88] and the central terminals (and postsynaptic targets) of several classes of functionally-distinct cutaneous primary afferents [24, 26, 69–71]. These show that virtually all axon terminals that form axoaxonic synapses show immunolabelling for GABA, and most (but not all) also label for glycine, while inhibitory axodendritic synapses are typically formed by boutons that contain glycine, GABA, or both transmitters. These anatomical observations support earlier reports from electrophysiological studies that presynaptic inhibition is mediated through activation of GABA_A_ (and possibly GABA_B_) receptors on the central terminals of primary afferents [57, 61, 63, 89], whereas postsynaptic inhibition can be mediated by either GABA or glycine, or the co-transmission of both [65, 84, 90]. Some inhibitory axon terminals form synaptic triads with the central terminals of primary afferents and dendrites that are themselves postsynaptic to the primary afferent [91–95], and these are likely to provide strict control over the passage of afferent input to postsynaptic dendrites by mediating both pre- and postsynaptic inhibition simultaneously. Most of the axon terminals involved in these triadic arrangements contain both GABA and glycine [24, 26, 69–71], and it is likely that while both transmitters are released at the axoaxonic and axodendritic synapses formed by these boutons, glycinergic inhibition only operates at the axodendritic synapses, given the absence of functional glycine receptors on primary afferents.

## Cellular Basis and Behavioral Consequences of Spinal Disinhibition

The importance of spinal inhibition in somatosensory processing was demonstrated in studies where strychnine and bicuculline (glycine and GABA_A_ receptor antagonists, respectively) were administered via intrathecal routes in rats [16]. This resulted in “a dose-dependent organized agitation response to light tactile stimulation,” which resembled tactile allodynia, a symptom reported by up to half of patients with neuropathic pain [96]. Tactile allodynia is often resistant to treatment, meaning that developing novel, more effective therapies presents a pressing clinical need [97]. Subsequent studies supported these initial findings [98–100], and the selective loss of spinal inhibition (spinal disinhibition) has been identified as an important contributor leading to the development of central sensitization and pathological pain [101–103]. Precisely how peripheral nerve injury induces spinal disinhibition, and the resultant effect this has on the activity of spinal circuits, remains a topic of considerable interest and debate (see reviews 104, 105, 106, 107, and 108). One of the most highly contested hypotheses proposes that peripheral nerve injury leads to selective loss of inhibitory interneurons in laminae I–III of the spinal dorsal horn through apoptosis [104–108]. However, a series of detailed anatomical studies have challenged these views, finding no loss of either GABA- or glycine-immunoreactive neurons in animals that had undergone partial peripheral nerve injuries that resulted in signs of neuropathic pain and showing that apoptosis in the dorsal horn after nerve injury was confined to microglia [8, 109, 110]. Although it has been shown that glutamic acid decarboxylase (the enzyme responsible for GABA synthesis) and mRNA encoding for the GAD65 isoform is down-regulated following nerve injury [107, 110, 111], other studies report no reduction of GABA levels in axon terminals of inhibitory interneurons from laminae I and II in the same partial nerve injury model [27].

Given that a loss of inhibitory interneurons and (or) a loss of GABA levels in the spinal dorsal horn remain topics of debate, other alternative mechanisms have been proposed to explain heightened excitability of dorsal horn circuits in conditions of neuropathic pain. One hypothesis implicates the downregulation of the chloride ion transporter KCC2 following peripheral nerve injury [112–115], brought about by the release of brain-derived neurotrophic factor (BDNF) from axotomized afferents [116, 117]. A reduction of KCC2 leads to disruption in chloride equilibrium, and this reduces the efficacy of inhibition mediated by the release of GABA and glycine in pain-transmitting neurons, whereas others have proposed that the reduced excitability of inhibitory interneurons and/or loss of their synaptic inputs are additional contributing factors [118–120].

Although the loss of spinal inhibition has been shown to allow A-LTMR afferent input to activate pain circuits in lamina I [121, 122], the route(s) through which this is achieved is/are poorly understood. Two distinct circuits through which this is achieved have been proposed, although both are gated by inhibitory PV-expressing interneurons (iPVINs) and involve the aberrant recruitment of vertical cells [60, 123]. Vertical cells have been proposed as likely candidates to facilitate the recruitment of pain circuits following A-LTMR activation given that their dendrites extend into lamina III and receive inputs from myelinated afferents, and their axon arborizes in lamina I to synapse on projection neurons that relay information to the spinoparabrachial nucleus [13, 124, 125]. PVINs are found primarily in the inner part of lamina II and in lamina III [126, 127], and most of these co-express both GABA and glycine [128]. These interneurons are known to play a crucial role in gating A-LTMR input into the spinal dorsal horn, given that selectively ablating them in naïve mice induces allodynia-like responses to mechanical stimuli, whereas chemogenetic activation of these cells in allodynic mice alleviates their mechanical hypersensitivity [123]. PVINs have been shown to be a source of axoaxonic inputs on to the central terminals of A-LTMRs [50, 60], and of axodendritic synapses on to several classes of interneuron populations known to receive direct input from myelinated afferents including vertical cells, interneurons that express the γ-isoform of protein kinase C (PKCγ), and other PV-expressing cells [124, 129]. The co-release of GABA and glycine at synapses formed by iPVINs initiates two types of inhibition (GABA-mediated presynaptic inhibition at axoaxonic synapses, and postsynaptic inhibition resulting from the action of both GABA and glycine), and supports the view that both transmitters play distinct roles in segregating A-LTMR afferent input from pain circuits and underlie their involvement in the development of different aspects of mechanical hypersensitivity [98, 130–133]. The most direct impact of losing inhibition mediated by PVINs would be the simultaneous reduction in presynaptic control of A-LTMRs and postsynaptic inhibition of vertical cells, allowing innocuous tactile inputs to activate lamina I pain circuits [60]. An additional consequence of losing PVIN-mediated inhibition is the loss of postsynaptic inhibitory drive to PKCγ interneurons [123]. These cells play an important role in neuropathic pain [134] by relaying A-LTMR information to pain circuits via transient central cells when glycinergic inhibition is compromised [41, 131, 132], and is brought about when PVIN-mediated inhibition is reduced after peripheral nerve injury [123].

Precisely how peripheral nerve injury leads to a loss of PV cell-mediated inhibition has yet to be established. There is no apparent loss of PV interneurons [60, 123], but whether the axons of these cells disconnect from their principal synaptic targets is yet to be resolved. What has become apparent is that some of the functional properties of PVINs change following peripheral nerve injury [60]. For example, the amplitude of current injection needed to maintain tonic firing for the entire stimulus in tonic-firing PV cells was significantly higher ipsilateral to the nerve injury than for the contralateral side, and the current-frequency relationship for action potential discharge was also significantly lower on the ipsilateral side. These changes are likely to result in a reduction of PV cell-mediated inhibition. An earlier targeted electrophysiological study of inhibitory interneurons in a GAD67::eGFP mouse line reported an impaired excitatory drive to GABAergic neurons after nerve-injured mice [135] but no change in either the excitability or discharge properties of these neurons [118, 119]. When similar experiments were conducted in a PV^Cre^; Ai9 mouse line, no change in excitatory drive to PV cells was seen, but distinct differences in the excitability and action potential firing patterns of these interneurons were reported [60]. These findings suggest that subtle physiological differences may become apparent when discrete subpopulations of inhibitory interneurons are targeted specifically.

## Future Directions—Novel Targets

One approach in helping to develop new therapies to tackle chronic pain states is to establish the functional significance of discrete neuronal populations in the spinal dorsal horn and then determine precisely how their associated anatomical features or electrophysiological properties change under pathological conditions. Experiments involving topical application of specific neurotransmitter receptor antagonists in freely moving animals were the first to establish the importance of spinal inhibition in somatosensory processing [16, 98–100]. These were followed by a series of *in vitro* electrophysiological studies where the properties of interneuron populations were recorded under normal or chronic pain conditions [42, 43, 107, 119], and more recently, where the activity of relatively large populations of cells was manipulated *in vivo* to determine [131–133, 136, 137]. Given recent technological advances and a better understanding of neurochemically distinct populations of spinal interneurons, we now have the unprecedented means of targeting and manipulating subpopulations of inhibitory interneurons with great precision [5, 11, 12, 36, 138].

**Fig. 4:**
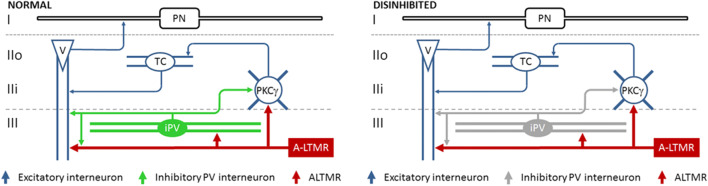
The role of inhibitory parvalbumin-expressing interneurons in gating low-threshold tactile input. Under normal conditions, inhibitory parvalbumin-expressing interneurons (iPV, green) mediate presynaptic inhibition of A-LTMR input (red) and postsynaptic inhibition of both vertical cells (blue, V) and PKCγ-expressing interneurons (blue, PKCγ). Peripheral nerve injury results in a reduction of iPV excitability (iPV, grey), leading to spinal disinhibition. The loss of iPV-mediated inhibition allows A-LTMR input to activate vertical cells directly, and through a polysynaptic route incorporating PKCγ-expressing interneurons and transient central cells (TC, blue). Under these conditions, vertical cells can relay A-LTMR input activate projection neurons (PN, black) in lamina I and recruit pain circuits. Based on references 48, 60, 123, 125

For example, manipulating the activity of PV interneurons specifically in both naïve and nerve-injured mice *in vivo* has been instrumental in establishing their role in setting mechanical thresholds [123]. By determining that the inhibition mediated by these cells play important roles in gating both A-LTMR input directly, and the relay of information through vertical cells, we find that they are uniquely placed to exert significant influence on the segregation of innocuous tactile information from pain circuits [50, 60]. When PV cell-mediated inhibition is lost, the disinhibition on A-LTMRs will likely lead to increased recruitment of several excitatory interneuron populations [139–143], and the aberrant recruitment of the circuits they contribute to in turn may underlie the difficulties we have faced to-date in developing effective therapies to manage chronic pain states (Fig. 4). Simply targeting one spinal circuit may not be sufficient to alleviate chronic pain, but since disinhibition of afferent input is believed to contribute to the development of several chronic pain states [17] and lies upstream of these spinal circuits, re-establishing presynaptic control of A-LTMRs in chronic pain states may be a more effective strategy.

To achieve this selectively with pharmacological approaches may be challenging given the widespread distribution and heterogeneity of GABA_A_ receptors in primary afferents and spinal dorsal horn neurons [79, 144], but by re-engaging specific neuronal populations and their outputs, rather than activating these receptors globally, it may be possible to restore normal function in experimental animal models with minimum additional consequences. Inhibition mediated by PV neurons is necessary to segregate A-LTMR input from pain circuits, and disinhibition of the circuits they serve is a significant contributor to the development of mechanical hypersensitivity. The loss of PV cell-mediated inhibition does not result from the death of these cells, but the reported changes in intrinsic electrophysiological properties of these cells imply that specific channelopathies within PV interneurons may be an important factor in the development of tactile allodynia. Although the precise mechanisms underpinning the changes in firing patterns and excitability seen in PV interneurons have yet to be determined, hyperpolarization-activated cyclic nucleotide–gated (HCN) channels are one of many possible targets given that they have been implicated in many pathological conditions including neuropathic pain [145–147]. HCN channels play critical roles in setting action potential firing patterns, and PV cells are known to show a high prevalence of I_*h*_ subthreshold currents and are enriched in both HCN1 and HCN4 subunits [50, 77]. It is tempting to speculate that changes in the properties of these channels in PV interneurons may contribute to the altered properties of these cells in chronic pain states. For example, a downregulation in HCN1 subunit expression (which confer faster kinetics on HCN channel complexes) in iPVINs, coupled with an increased expression of the more slowly conducting HCN4 subunits, could contribute to the reduced excitability seen in these cells after nerve injury [60]. Although changes in HCN subunit expression in distinct dorsal horn neuron populations have yet to be reported following peripheral nerve injury, it has been shown that mRNA for both HCN1 and HCN2 is markedly decreased in dorsal root ganglion neurons following axotomy [145, 148]. Should spinal interneurons undergo similar changes, restoring normal HCN subunit expression in these cells could re-establish spinal inhibition and alleviate the mechanical hypersensitivity seen in pathological conditions. The widespread expression of various HCN channel complexes in non-neuronal tissue poses significant problems when antagonists are administered systemically, but by developing intersectional strategies to target these channels in spinal interneurons specifically, it is now possible to study their contribution to the development of chronic pain states in a variety of animal models. The recent advances made in defining, targeting, and manipulating discrete neuronal populations now provide us with unprecedented means of studying distinct components of neuronal circuits in animal models, and this generates real hope that more effective treatments for treating chronic pain will soon be available.

## Electronic Supplementary Material

ESM 1 (PDF 1224 kb)

ESM 2 (PDF 1224 kb)
